# The Effects of Group-Based versus Individual-Based Tai Chi Training on Nonmotor Symptoms in Patients with Mild to Moderate Parkinson's Disease: A Randomized Controlled Pilot Trial

**DOI:** 10.1155/2017/8562867

**Published:** 2017-07-24

**Authors:** Jing Hui Yang, Ya Qun Wang, Sai Qing Ye, You Gen Cheng, Yu Chen, Xiao Zhen Feng

**Affiliations:** ^1^Department of Rehabilitation Medicine, Tongde Hospital of Zhejiang Province, Hangzhou, China; ^2^Department of Rehabilitation Medicine, West China Hospital of Sichuan University, Chengdu, China; ^3^Wuxi Tongren International Rehabilitation Hospital, Wuxi, China

## Abstract

**Objective:**

To compare the effects of group-based and individual-based Tai Chi training on nonmotor symptoms in patients with mild to moderate Parkinson's disease.

**Design:**

Randomized controlled pilot study.

**Methods:**

36 community-dwelling patients with Parkinson's disease (PD) were randomly assigned to either group-based training group (*n* = 19) or individual-based group (*n* = 17). Both groups received same content of Tai Chi training 3 times a week for 13 weeks. Participants were also asked to perform home exercises daily. The Non-Motor Symptoms Scale was used to assess global nonmotor symptoms change. Sleep quality, depression, and cognition were evaluated by Parkinson's Disease Sleep Scale, Hamilton Depression Scale, and Beijing version-Montreal Cognitive Assessment, respectively. Home exercise compliance was recorded.

**Results:**

There was no significant difference between two groups at baseline. After 13 weeks, there were no statistical significance between two groups. However, the within-group effect was different. Participants in group-based and individual-based groups showed a significant improvement on global nonmotor symptoms (*P* < 0.001, *P* = 0.004) and sleep (*P* < 0.001, *P* < 0.001). But only group-based training patients presented a significant improvement in cognitive impairment compared with baseline (*P* = 0.002, *P* − 0.116). For depression, no group gained a significant improvement(*P* = 0.123, *P* = 0.170). Group-based participants had a higher home-exercise compliance rate (HeCR) than individual-based participants did (*P* = 0.019), and HeCR showed a moderate correlation with MoCA-BJ and NMSS scores changes in this study.

**Conclusion:**

Group-based Tai Chi training is considered to be a more effective and a more labor-saving method in the clinical settings, and patients tend to have a higher compliance rate in their home exercise program. This study is registered with ChiCTR-IPR-17010388.

## 1. Introduction

Idiopathic Parkinson's disease (PD) is a common neurodegenerative disorder affecting worldwide 1% of people over 55 years of age [1]. While PD is most commonly associated with motor symptoms, there are numerous nonmotor symptoms (NMS) associated with the condition as well, such as sleep disturbance, cognitive decline, anxiety, and depression [2]. NMS are found not only in the advanced stage but also in the early stage of the disease and may precede the onset of motor symptoms by several years [3]. Furthermore, some recent studies showed that NMS in PD patients could substantially reduce the quality of life to a greater extent than the motor symptoms [4, 5]. NMS in PD patients, especially sleeping disturbance, mood changes, and dysautonomia, can be caused by not only pathological alterations due to the disease itself but also the traditional dopaminergic treatments [6]. Therefore, a wide range of nonpharmaceutical therapies have now been delivered to PD patients. Many studies of different exercise models have been delivered to PD patients to evaluate the therapeutic effects on nonmotor symptoms, but the results are still controversial. In 2013, Shulman et al. [7] compared the efficacy of treadmill exercise and stretching and resistance exercise in improving gait speed, strength, fitness, and also nonmotor symptoms for patients with PD. Even though the three types of exercise demonstrated varied effectiveness in motor symptoms, no benefits were found in any nonmotor symptoms for any exercise group, including depression, fatigue, and quality of life. Some mind-body exercises were also applied to treat nonmotor symptoms in PD patients. Sharma et al. [8] reported a positive trend in the improvement of depression level after 24 sessions of yoga training. However, Schmitz-Hübsch et al. [9] showed insignificant findings of depression level after total of 6-month intervention of Qigong exercise.

Tai Chi is another option and seems to be a better choice. As a mind-body exercise intervention, Tai Chi consists of a series of slow and smooth movements with strict and mindful breathing and body control. A large-scale randomized trial conducted by Li and his colleagues in 2012 showed that Tai Chi training had better performance in reducing balance impairments, improving functional capacity, and reducing falls when compared to resistance training and stretching [10]. Besides improving motor function, Tai Chi had also been proved effective in relieving stress and thus improving the quality of life [11]. A recent study by Nocera et al. showed the positive changes on cognitive function and psychological wellbeing of patients with PD following 16-week Tai Chi exercise [12].

In China, the number of individuals with PD over age of 50 was approximately two million in 2005, and the population was expected to be nearly 4.94 million by the year 2030 [13]. The most frequently used approach is individual-based training, in which patients receive 1-on-1 training from a physiotherapist. With the rising number of PD patients and limited fiscal resources in the public health sector, China might face the dilemma of shortage of therapists for PD patients in near future. Furthermore, some researchers believed that different circumstances, such as a natural scenery, group-based exercise, and presence of family member, might have a positive training effect on NMS in PD patients [14]. Group settings could possibly contribute to aspects such as exercise frequency and involvement because of the peers' encouragement and the competition. So, we hypothesized that group-based training, as a more labor-saving option, may be an appealing approach if it can be proved to produce similar or even better outcomes. To date, there is no study that had directly compared the effects of group-based and individual-based Tai Chi training in this population.

This randomized controlled study aimed to compare the effects of group-based and individual-based Tai Chi training on NMS in PD patients. As this is a pilot study, the study designers also evaluated the compliance with home exercise during the study. Such information could provide useful insight in further development and implementation of Tai Chi training for the PD patients.

## 2. Methods

### 2.1. Ethics Statement

This pilot study was conducted at the Department of Rehabilitation Medicine of Tongde Hospital of Zhejiang Province in Hangzhou, China, between February 2016 and October 2016. It was conducted in strict accordance with the protocol approved by the Ethics Committee of Tongde Hospital of Zhejiang Province (Hangzhou, China). Written informed consent was obtained from all subjects (or their guardians) prior to enrolment.

### 2.2. Study Design

A randomized controlled pilot intervention trial was undertaken to evaluate the effects of 13-week Tai Chi training on NMS in patients with mild to moderate Parkinson's disease and compared the two delivery methods: group-based and individual-based training. The outcome assessors were blinded to the group allocation, making this a single-blinded study.

### 2.3. Subjects

A convenience sample of community-dwelling participants with idiopathic PD was recruited for this study. All the subjects recruited in this study had the membership of the* Parkinson's Disease Club*, which was established by Rehabilitation Medicine Department of Tongde Hospital in 2013. The club includes 113 registered PD patients, who regularly gather two times per month. PD patients in this club received suggestions about medication, diet, and exercise from doctors and therapists of the department. Psychological counselling is also provided by psychologists.

Evaluations and complete neurological examination were performed by experienced neurologists. Participants were included if they (1) were diagnosed of idiopathic PD by UK PD Brain Bank criteria, (2) had a disease rating of stages I to III on the Hoehn and Yahr (H&Y) scale, (3) were between 50 and 75 years old, (4) had stable medication usage, (5) could perform a whole circle of Tai Chi exercise without assistance, and (6) were willing to have Tai Chi exercise. Participants were excluded if they (1) had any history or evidence of neurological deficit other than PD, (2) had dementia (determined by a Beijing version-Montreal Cognitive Assessment less than 17), (3) suffered from significant depression (determined by a Hamilton Depression Scale larger than 20), (4) had somatic disease that could have a potential effect on NMS (e.g., pain syndromes, advanced diabetes mellitus), or (5) were on medications affecting balance or attention.

**Figure 1:**
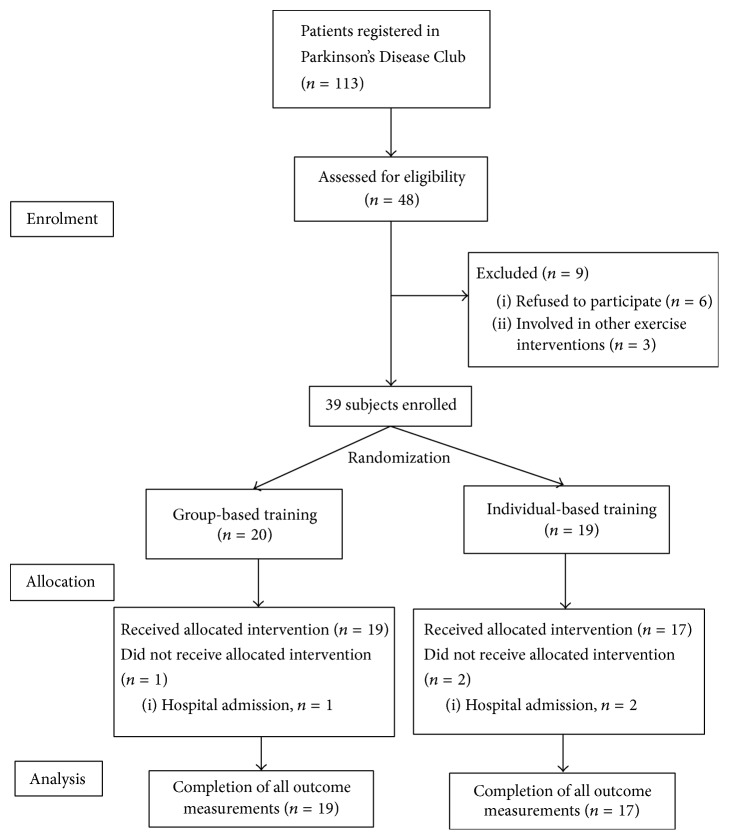
Patient flow diagram.

A total of 48 PD patients in the Parkinson's Disease Club met the criterion (Figure 1). Three of the 48 PD patients underwent another clinical study and were excluded from this study. The remaining patients were provided with detailed information regarding the purpose, procedure, interventions, and potential risks of the study by the research personnel. Six of the 45 PD patients refused to participate in the study because of the risk of fall or other considerations. Thus, 39 patients enrolled in this study.

### 2.4. Randomization

To ensure that the number of subjects in each group was approximately equal, each patient was given a random number following a random number table and ordered by their assigned numbers. The patients were then assigned to groups by taking the first patient in the order list for group-based group (GbG), the next patient for the individual-based group (IbG), and so on until all were assigned. The randomization procedure was performed by a research therapist who was not involved in the assessment of the subjects. There were 20 patients in GbG and 19 patients in IbG at the start of the study.

### 2.5. Intervention

The Tai Chi classes were delivered by a 7-year experienced Tai Chi instructor who was specialized at 24-form Yang Style Tai Chi [15]. This style of Tai Chi is easy to learn and is the most popular form of Tai Chi in China. It emphasizes diagonal weight shifting, awareness of body position, and breathing. Every participant was taught in a one-on-one fashion to ensure they all understood and performed well before the beginning of the study.

Both groups received 39 sessions of 24-form Yang Style Tai Chi for 40~45 minutes each time, three times a week for 13 weeks. At the first 2 weeks, the training time was 20–25 minutes per session due to the bad endurance in these subjects, and the subjects could have a break when they reported any uncomfortable feelings, such as dizziness, tiredness, or nausea.

The patients to therapist ratio was 6~7 : 1 for the group-based training and 1 : 1 for individual-based training at the beginning. To maintain the consistency of the treatment approach, both group-based and individual-based training were conducted by the same Tai Chi instructor.

Every subject was suggested to do Tai Chi exercise at home every day (except for the days on which hospital training sessions took place) during the study period. A diary was used to record the home exercise date and duration for every patient. The home exercise content was the same as what they did in the hospital, but the patients did not need to repeat the whole session of the exercise. The home Tai Chi exercise would take approximately 20 to 25 min to be completed. The patients and their caregivers were provided with audiovisual material, and the caregivers were asked to be present to prevent falls and supervise accurate movement during the home exercise.

### 2.6. Outcome Measures

*Nonmotor Symptoms*. NMS may present in varied manifestations in PD patients. Chaudhuri et al. [15] have developed the Non-Motor Symptoms Scale (NMSS) for PD patients' evaluation. This scale is a reliable and valid tool for assessing the progress or potential response to treatment of NMS in PD patients. NMSS contains nine dimensions: cardiovascular (2 items), sleep/fatigue (4 items), mood/cognition (6 items), perceptual problems (3 items), attention/memory (3 items), gastrointestinal (3 items), urinary (3 items), sexual function (2 items), and miscellany (4 items). Score for each item is based on a multiple of severity (from 0 to 3) and frequency scores (from 1 to 4), and a higher score indicated more severer symptoms. NMSS was used to evaluate the global NMS changes in all subjects before and after the Tai Chi intervention in this study.

According to Song and other researchers, sleep dysfunction, mood problem, and cognition disorder are the most prevalent NMS in China's PD patients [16, 17], and these NMS are closely associated with the Health-Related Quality of Life of PD patients [18–20]. Therefore, we decided to use more specific measurement tools to assess these symptoms in these subjects. Sleep quality was assessed by Parkinson's Disease Sleep Scale (PDSS) [21]. Hamilton Depression Scale (HAMD) was used to assess the depression in PD patients. For cognitive impairment evaluation, Beijing version-Montreal Cognitive Assessment (MoCA-BJ) was used because it is more sensitive than the MMSE for detecting early cognitive decline and impairment change in nonmemory dysfunction in Chinese patients [22].

Each subjects' nonmotor symptoms were evaluated within five days of the initiation of the study and again within five days of the termination of the 13-week intervention. Both groups assessments were performed by the same independent assessor, who was an experienced physiotherapist and was blinded to the group assignment. The patients and their caregivers were instructed not to inform the assessor of the group allocation. The outcome assessor was also instructed not to ask the subjects which group they belonged to. 

*Home Exercise Compliance.* A diary was used to record home exercise date and duration, and only when the exercise time was more than 20 min/day would it be regarded as ONE home exercise day. The home exercise compliance rate (% days) was calculated using the following formula: (number of days of home exercise participation) *∗* 100%/(total number of days).

### 2.7. Statistical Analysis

Descriptive statistics are reported as mean ± SD. Between-group differences in demographic and baseline variables were tested with a chi-square test for categorical variables and an independent *t*-test for continuous variables.

Independent *t*-test was used to compare the change scores between the groups. Paired *t*-test was used to examine within-group changes from baseline to 13 weeks. The Pearson's correlation coefficient was used to explore whether the changes of total NMSS scores and other measurement scores were associated with the home exercise compliance rate. All the statistical analyses were performed using the Statistical Package for the Social Sciences (SPSS Inc., Chicago, IL, USA) software program version 21.0. The level of significance was set at 0.05 (2-tailed). In addition, post hoc power analysis was performed. The effect size and power were calculated using G^*∗*^Power (Faul and Erdfelder, Bonn University, Germany, 1992) statistical software version 3.1.9.2.

## 3. Result

48 PD patients were eligible for the study, 39 initiated the study, and 36 patients completed both pre- and postassessments. One participant and 2 participants withdrew from the group-based and individual-based group, respectively, because of hospital admission. As such, 19 group-based training participants and 17 individual-based training participants completed the study. No adverse event was reported during the study period.

There were no statistically significant differences between the 2 groups at baseline for all measures, including age, the proportion of male and female, height, weight, disease duration, age at onset, or the H&Y scale and UPDRS Total score (Table 1).

Pre- and postintervention results of the outcome measures were depicted in Table 2. After 13 weeks of Tai Chi training, both group-based and individual-based groups showed a significant improvement on global nonmotor symptoms (NMSS score: *P* < 0.001, *P* = 0.002) and sleep (PDSS score: *P* < 0.001, *P* < 0.001). However, only group-based group presented a significant improvement in cognitive impairment (MoCA-BJ score: *P* = 0.002, *P* = 0.116) after the intervention. And, for depression, no group gained a significant improvement after the intervention (HAMD score: *P* = 0.123, *P* = 0.170). Even though the mean changes of all outcome measures in group-based group were bigger than individual-based group, there were no statistical significance between two groups. For instance, the mean changes in MoCA-BJ score in group-based group were much bigger than individual-based group (1.63 versus 0.58), but no statistical difference (*P* = 0.081) showed. Similar results were found in NMSS score (9.94 versus 6.64, *P* = 0.282), PDSS score (10.73 versus 8.29, *P* = 0.506), and HAMD score (0.84 versus 0.76, *P* = 0.918) as well.

**Figure 2:**
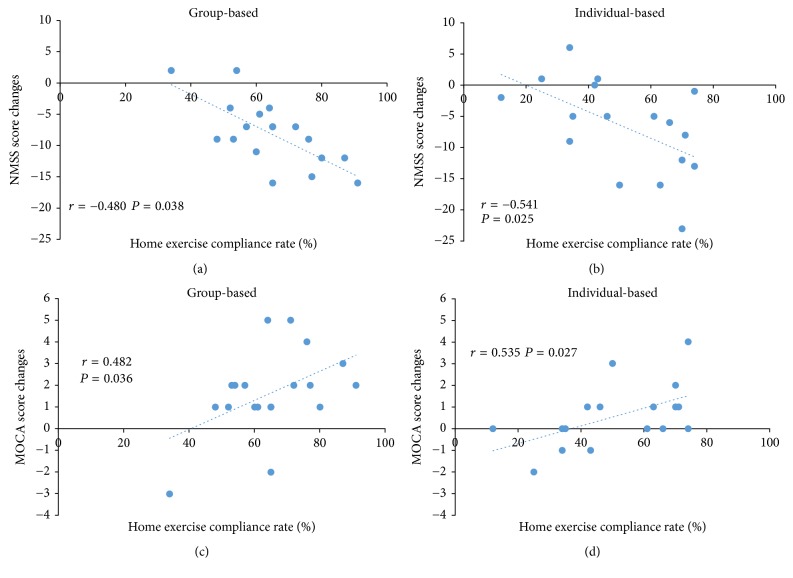
Significant correlation between the home exercise compliance rate (HeCR) and the changes in NMSS total scores and MOCA scores: (a) correlation between HeCR and the changes in NMSS total scores in group-based group; (b) correlation between HeCR and the changes in NMSS total scores in individual-based group; (c) correlation between HeCR and the changes in MOCA scores in group-based group; (d) correlation between HeCR and the changes in MOCA scores in individual-based group.

Home exercise compliance was significantly different between 2 groups (group-based: mean 64.84% (SD 13.99%); individual-based: mean 51.17% (SD 19.12%), *P* = 0.019). There was also a moderate correlation between the home exercise compliance rate and the change in MoCA-BJ scores and NMSS total scores following either group-based group (*r* = 0.482, *P* = 0.036; *r* = −0.480, *P* = 0.038) and individual-based group (*r* = 0.535, *P* = 0.027; *r* = −0.541, *P* = 0.025) (Figure 2). However, the data showed no significant correlation between home exercise compliance rate and the changes in PDSS score (*r* = 0.380, *P* = 0.109; *r* = 0.195, *P* = 0.453) and HAMD score (*r* = 0.029, *P* = 0.907; *r* = 0.220, *P* = 0.397) following two groups.

### 3.1. Post Hoc Power Analysis

The estimated effect size for NMSS, PDSS, HAMD, and MoCA-BJ was 0.40, 0.40, 0.04, and 0.72. These values represent a small-to-medium effect size. With an alpha value of 0.05 and a sample size of 19 and 17 subjects in each group, the corresponding statistical power was calculated as 0.22, 0.21, 0.05, and 0.55, respectively.

## 4. Discussion

Nonmotor symptoms in PD patients can present at the very early stage and are highly impactful on quality of life. To date, most studies have investigated motor outcomes following Tai Chi exercise in patients with Parkinson's disease and many have demonstrated improvements [23]. However, most have ignored the potential additional benefits to NMS of this body-mind interaction activity. In this study, we compared group-based and individual-based Tai Chi training on NMS in patients with PD. After 13 weeks' intervention, the difference between two groups was not big, but we can still dig some interesting findings.

### 4.1. Group-Based versus Individual-Based Tai Chi Training

This present study compared the effects of 13 weeks' group-based and individual-based Tai Chi training on NMS in patients with mild to moderate PD. The results showed that both groups improved significantly in global NMS and sleep quality when compared to baseline. Even though the differences between two groups failed to reach the statistical significance level, the score changes in group-based group were larger than those in individual-based group. The possible explanation for the similar outcomes is that each approach we used in this study has a positive impact on NMS (except for depression), and the limited study duration could not show the superiority of the compared approaches. But for home exercise compliance rate (HeCR), group-based patients seem more likely to accomplish the home exercise (assigned by the study designer) than individual-based patients do. It may be impossible to distinguish whether the better outcomes in group-based group are because of the group setting itself or the higher home exercise frequency. However, HeCR showed significant correlation with the global NMS and cognitive function. A higher HeCR may lead to a better outcome in a consistent way. So, we suppose that if we extend the study duration in the future, such as six months or one year, the patients in group-based setting may gain more obvious improvement on nonmotor symptoms.

The group-based patients have a higher home exercise frequency. The significantly higher HeCR may be due to more support and competitive motivations from his or her peers. First, the group-based training provides subjects more opportunities of social interaction and mind support. Secondly, group training makes participant more competitive, and this motivates them to perform better. Then, once they have a better performance in front of his or her groupmates, they may get more support and praise from the group. This virtuous circle may further encourage the subjects' participation in the training and home exercise [14]. In addition, a previous study found that social support networks were low in older adults compared with younger individuals, partly due to issues such as peers becoming less active and family fearing accidently falling or other injuries [24]. Thereby, group-based training could be more beneficial to the participants, especially for elders.

As a traditional Chinese exercise, Tai Chi is considered as an exercise requiring high level of attention and memory to learn and complete. In this study, we measured cognitive function changes before and after intervention. Both groups presented an increase of MoCA-BJ scores, but only group-based participants showed a significant improvement. We think the reason of this result is mainly because of the participant in group-based group did more Tai Chi exercise at home. Another explanation is exercise environment. According to Berman et al. [25], the interaction with natural environment could provide more cognitive function control when compared with urban environment. In this study, the participants usually chose community garden as the place to accomplish home exercise, which gives them more chance to expose themselves to the nature. This result also reminds us that if we want the patients to receive more cognitive control we should think about replacing the hospital training at a more natural environment in the future.

As for depression, neither of the group showed a significant improvement. Similar results for depression recovery were reported in studies using different type of exercise in PD patients. In 2013, Shulman et al. [7] compared the efficacy of 3 types of exercise (high intensity, low intensity, and resistant training) in PD patients. After 3-month intervention, all the participants in 3 groups did not show a significant improvement on depression. The same results could be seen in a tango exercise [26] and a dance intervention study [27] too. But a good result was shown in a formal exercise study [28] and a Nordic Waling program [29]. The participants in these 2 trials presented a significant improvement after intervention. The reasons for the conflicting results may due to the different exercise type, different measurement tools, and the study design, including training frequency and duration. Moreover, there was still not enough evidence on Tai Chi training in PD patient's depression issues.

### 4.2. Group Training and Home Exercise

Receiving exercise intervention in a group context may be particularly healthy for PD patients, which is at considerable risk of daily life and health care stigmatization and social isolation [30]. Also, PD patients' home exercise compliance is another issue that should be considered. In this study, we test the efficacy of group training plus home exercise program; the results showed a positive effect while compared to individual training. We believe that group training is a better option when facing the increasing number of clients, limited staffing,and budgetary cuts, the common issues nowadays. Besides, considering the inconvenient transportation for PD patients and the busy schedule of their caregivers, the implementation of home exercise program is an alternative method to increase the exercise frequency and duration without cost to the healthcare system.

### 4.3. Review Studies of Different Approaches on NMS in Parkinson's Disease

Some randomized controlled studies have examined the effect of different kind of nonpharmaceutical therapy on NMS in patients with PD. In a multicentre, randomized controlled clinical trial, Sturkenboom et al. [31] compared the efficacy of 10 weeks of home-based occupational therapy with usual care with no occupational therapy for patients with PD. The results showed that even though OT group gained a better self-perceived performance on prioritised activities (Canadian Occupational Performance Measure performance scale) at 3- and 6-month follow-up, the mean changes between two groups in quality of life (Visual Analogue Scale for QoL), fatigue (Fatigue Severity Scale), and depression (Beck Depression Inventory) were not significant. By contrast, Modugno et al. [32] found that PD patients (Hoehn and Yahr stages 2–4) who had undergone a theatre workshop rehabilitation program (6-h daily sessions, for a total of ~18 h/months, for 3 years) that was a combination of exercising basic skills and theatre performance skills (recreation of stage behaviours and emotions that could occur in real life) showed a very quick and significant improvement in the mood, quality of sleep, and perception of social support (as assessed by Hamilton Depression Rating Scale, Epworth Sleepiness Scale, and PDQ39 social support scale) compared with PD patients in the physiotherapy group. The authors believe that these remarkable results may be due to the combination of movements and stimulation of different sensory pathways and emotional involvement. In order to be an eligible “actor,” PD patients have to control their movements and emotions simultaneously. In addition, theatre therapy asked patients to interact continuously, so they are forced to socialize. These features make theatre an ideal playground to motivate patients deeply. Some impressive benefits on NMS can also be found in a Nordic Waling study we mentioned before [29]. In this study, PD patients showed a significant improvement on all NMS that measured, including global NMS (NMSS), fatigue (PFS-16), depression (BDI), and apathy (SAS), after 12-week NW exercise. A study conducted by Foster et al. [33] in 2013 demonstrated that a community-based tango dance program (every PD patient was paired with a healthy caregiver as a group) could significantly increase the current activity participation, activity retention, and numbers of New Social activities after 12 months' intervention. Taken together, these findings indicate that we should pay more attention to other conditions when treating NMS in PD patients. These conditions may include natural environment (Nordic walking), group or paired exercise (Tai Chi, tango, and theatre training), novel and attractive intervention methods (theatre training), and other circumstances that may potentially increase the interactions with others or the activity participation.

## 5. Conclusion

Both group-based and individual-based Tai Chi training are effective in improving nonmotor symptoms in patients with mild to moderate Parkinson's disease. Group-based Tai Chi training is considered a more effective and a more labor-saving method in the clinical settings, and patients tend to have a higher compliance rate in their home exercise program. This may provide some insights for method of delivering Tai Chi training to PD patients in the future.

## Figures and Tables

**Table 1:** Baseline characteristics of participants in group-based and individual-based groups.

	Group-based group (*n* = 19)	Individual-based group (*n* = 17)	*P* value
Age	62.94 (5.45)	64.23 (5.72)	0.415
Gender (M/F)	11/8	9/8	0.765
Height (cm)	171.37 (5.63)	173.41 (4.14)	0.232
Weight (kg)	67.47 (5.95)	69.29 (6.29)	0.594
Disease duration (years)	4.89 (2.20)	4.47 (1.54)	0.531
Age at onset	58.05 (5.67)	59.76 (6.48)	0.379
H&Y scale	1–3	1–3	0.827
UPDRS total	31.10 (6.54)	31.52 (5.10)	0.900

Numbers in parentheses designate standard error. H&Y scale, Hoehn and Yahr scale; UPDRS, Unified Parkinson's Rating Scale.

**Table 2:** Comparison of mean changes before and after intervention.

Outcome measure	Group	Time		Changes Mean (SD)	*P* value
		Baseline Mean (SD)	After 13 weeks Mean (SD)		
NMSS	Group-based	61.26 (19.86)	51.31 (20.72)	−9.94 (9.07)^*∗∗*^	0.000
	Individual-based	68.82 (25.31)	62.17 (22.47)	−6.88 (7.54)^*∗∗*^	0.002
	*P *value: GbG versus IbG	0.323		0.282	
PDSS	Group-based	97.42 (15.81)	108.15 (16.32)	10.21 (10.82)^*∗∗*^	0.000
	Individual-based	96.82 (20.35)	105.11 (20.22)	8.29 (4.77)^*∗∗*^	0.000
	*P* value: GbG versus IbG	0.923		0.506	
HAMD	Group-based	12.94 (3.59)	12.10 (3.05)	−0.84 (2.26)	0.123
	Individual-based	13.00 (3.35)	12.23 (3.68)	−0.76 (2.19)	0.170
	*P* value: GbG versus IbG	0.964		0.918	
MoCA-BJ	Group-based	21.26 (3.05)	22.89 (2.86)	1.63 (1.94)^*∗∗*^	0.002
	Individual-based	22.00 (2.78)	22.58 (2.51)	0.58 (1.46)	0.116
	*P* value: GbG versus IbG	0.456		0.081	
HeCR	Group-based		64.84 (13.99)^#^		
	Individual-based		51.17 (19.12)		
	*P* value: GbG versus IbG		0.019		

Numbers in parentheses represent the standard error. ^*∗∗*^*P* < 0.01 compared with baseline. ^#^*P* < 0.05 group-based group (GbG) compared with individual-based group (IbG) after 13 weeks.. NMSS, Non-Motor Symptom Scale; PDSS, Parkinson's Disease Sleep Scale; HAMD, Hamilton Depression Scale; MoCA-BJ, Beijing version-Montreal Cognitive Assessment; HeCR, home exercise compliance rate.

## Additional Points

*Limitations*. The study has several limitations. First, as this was a pilot study, the sample size was relatively small. The results should thus be interpreted with caution, as the post hoc power analysis shown was low. Secondly, the intervention duration was relatively short. We think the results may be different if we have a 6-month or longer intervention based on the HeCR. Thirdly, we did not include a nontreatment control group. Thus, even though both groups showed an improvement after Tai Chi training, we cannot exclude the possibility that the good results in both groups were the consequence of normal medication treatment during the treatment period.
